# Detection of *Leishmania* spp in silvatic mammals and isolation of *Leishmania* (*Viannia*) *braziliensis* from *Rattus rattus* in an endemic area for leishmaniasis in Minas Gerais State, Brazil

**DOI:** 10.1371/journal.pone.0187704

**Published:** 2017-11-27

**Authors:** Agnes Antônia Sampaio Pereira, Eduardo de Castro Ferreira, Ana Cristina Viana Mariano da Rocha Lima, Gabriel Barbosa Tonelli, Felipe Dutra Rêgo, Adriano Pereira Paglia, José Dilermando Andrade-Filho, Gustavo Fontes Paz, Célia Maria Ferreira Gontijo

**Affiliations:** 1 Grupo de Estudos em Leishmanioses, Instituto René Rachou, FIOCRUZ/MINAS, Belo Horizonte, Minas Gerais, Brazil; 2 Fundação Oswaldo Cruz Mato Grosso do Sul, Campo Grande, Mato Grosso do Sul, Brazil; 3 Departamento de Biologia Geral, Instituto de Ciências Biológicas, Universidade Federal de Minas Gerais, Belo Horizonte, Minas Gerais, Brazil; Taibah University, SAUDI ARABIA

## Abstract

Knowledge of potential reservoirs of *Leishmania* spp. in an anthropic environment is important so that surveillance and control measures can be implemented. The aim of this study was to investigate the infection by *Leishmania* in small mammals in an area located in Minas Gerais, Brazil, that undergoes changes in its natural environment and presents autochthonous human cases of cutaneous leishmaniasis (CL) and visceral leishmaniasis (VL). For the capture of the animals, Sherman and Tomahawk traps were used and distributed in the peridomicile of houses with reports of autochthonous cases of CL or VL. Six catches were carried out on two consecutive nights with intervals of two months during one year and samples of spleen, liver, tail skin, ear skin and bone marrow of the animals were obtained. Parasitological and molecular methods were used to detect the infection. Identification of the *Leishmania* species was performed by PCR RFLP*hsp70*. Twenty five animals of four species were captured: ten *Rattus rattus*, nine *Didelphis albiventris*, five *Cerradomys subflavus* and one *Marmosops incanus*. In the PCR-*hsp*70, five animals were positive (20%). The *Leishmania* species identified in PCR-RFLP*hsp*70 were: *Leishmania braziliensis* in *D*. *albiventris* (2), *C*. *subflavus* (1) and *R*. *rattus* (1) and *Leishmania infantum* in *R*. *rattus* (1). The highest positivity rate for *L*. *braziliensis* was obtained in the liver samples. The spleen was the only tissue positive for *L*. *infantum*. It was isolated in culture medium *L*. *braziliensis* from two samples (liver and spleen) of *R*. *rattus*. This is the first record of isolation of *L*. *braziliensis* from *R*. *rattus* in the southeastern region of Brazil. These results are relevant to the knowledge of the epidemiology of leishmaniasis in the region, mainly in the investigation of the presence of hosts and possible reservoirs of the parasite.

## Introduction

Leishmaniasis is considered the most complex and diverse of all vector-borne diseases with regard to ecology and epidemiology. More than 30 species of the genus *Leishmania* are recognized, infecting several species of hosts and reservoirs, and about 30 species of phlebotomine sand flies are incriminated as vectors of some *Leishmania* species [1,2]. In Brazil, species of the subgenus *Leishmania (Viannia)* and the species *Leishmania (Leishmania) amazonensis* are associated with human cases of cutaneous leishmaniasis (CL), a disease that affects the skin and in some cases can occur in the mucous membranes. *Leishmania (Leishmania) infantum* is the causative agent of visceral leishmaniasis (VL), a systemic disease that is potentially fatal to humans when appropriate treatment is not established. However, human and canine cases of leishmaniasis caused by *L*. *amazonensis* have been reported [3, 4, 5, 6].

In the Americas, more than 40 species of mammals have been infected by some species of *Leishmania*, but only some of these animals has the role of reservoir for the parasite. Until now, most of the studies that observed infection with *Leishmania spp*. in wild animals have been based on detection with molecular methods [7, 8], and there have been few reports of the isolation and characterization of the parasite [9, 10]. A detection of *Leishmania* spp. DNA in some species of mammal is not enough to consider this species as reservoir [11]. An animal found infected is a host of a parasite, whereas the term reservoir is more specifically defined as a system in which an infectious agent survives persistently [12]. So, a reservoir can be considered not just a single species of infected mammal, but a system which can include one or more species of animals responsible for maintaining the parasite in nature [11, 13, 14]. In reservoir systems, each mammalian species plays a role in maintaining the parasite, which means that these systems should always be considered on a restricted spatio-temporal scale and for each specific place and time [15]. The importance of these concepts to the control of american leishmaniasis is the fact that, in most cases, they are zoonoses that affect other mammals, and man is an accidental host [12].

In recent years, the interest by the scientific community in animals of synanthropic habits has increased, especially because leishmaniasis is increasingly being observed in urbanized areas [16]. The identification of the etiological agent circulating in endemic areas, as well as the knowledge of the role of the hosts-reservoirs, are important issues for the development of appropriate preventive measures to reduce the incidence of the disease.

In this study we investigated the occurrence of infection by *Leishmania* spp. in small mammals of an area of campo rupestre (Brazilian rupestrian fields) with consolidated anthropic occupation and where there have been autochthonous cases of VL and CL.

## Materials and methods

### Study area

The study was conducted in the locality of Casa Branca in the municipality of Brumadinho, Minas Gerais, Brazil. The population of Casa Branca is 2,591 and corresponds to 7.6% of the total population of the municipality of Brumadinho (20°08’36”S, 44°11’59”W), which has 33,973 inhabitants [17] and is located 49 km from Belo Horizonte, the capital of the State of Minas Gerais, Brazil.

### Animal collection

Small mammals were captured in nine peridomicile areas of residences from May 2013 to July 2014. The traps were distributed in the peridomicile of houses with human cases of CL and VL and dogs with VL reported in the municipality of Brumadinho between 2008 and 2011. The residences had areas of dense vegetation and poorly anthropized. Eight collecting campaigns were undertaken, one every two months, using thirty-six traps per campaign, including eighteen Sherman traps and eighteen Tomahawk traps. The traps were set between 10 and 20 meters away from the houses for two consecutive days per collection campaign and the total sampling effort was 576 traps/days (36 traps x 16 days). Authorization for sampling was granted by the Instituto Brasileiro do Meio Ambiente e dos Recursos Naturais Renováveis (IBAMA) number 12989–1.

### Samples

The study was regulated by the Brazilian Institute of Environment and Renewable Natural Resources (Instituto Brasileiro do Meio Ambiente e dos Recursos Naturais Renováveis—IBAMA), and registered under the license 12989–1. All procedures involving experimental animals were conducted according to the guidelines of the Brazilian College for Experiments with Animals (Colégio Brasileiro de Experimentacão Animal/COBEA—Law 11.794/2008).

The captured animals were euthanized and samples of liver, spleen, tail skin, ear skin and bone marrow were collected. Part of the samples were stored at -4°C in saline solution containing antibiotics (penicillin and streptomycin 100-200ųg/ml) for parasitological analysis and part stored at -20°C for molecular analysis. Species identification of the captured animals was based on the analysis of morphological characters and comparisons with specimens deposited in the Collection of Mammals of the Universidade Federal de Minas Gerais.

### Isolation of *Leishmania* in culture media

Tissue samples (liver, spleen, tail skin, ear skin and bone marrow from all animals collected) stored in saline solution were macerated and inoculated in NNN medium (Novy—MacNeal-Nicolle)/LIT (liver infusion tryptose) [18] supplemented with 20% fetal bovine serum (FBS) and associated antibiotics (penicillin and streptomycin 100-200ųg/ml). Tubes were maintained at 25°C±1°C in a B.O.D. incubator, examined weekly and considered positive when promastigotes of *Leishmania* were observed. Positive cultures were prepared for molecular analysis and deposited in the criobank of the Instituto René Rachou/FIOCRUZ.

### Molecular diagnosis

The extraction of DNA from all biological samples collected and isolates was carried out using the Puregene Cell and Tissue—QIAGEN kit following the manufacturers’ instructions. PCR was carried out targeting a fragment of the gene coding for heat shock proteins of 70 kilodaltons (*hsp*70) of *Leishmania* spp. using the primers for HSP70 for: 5’ GACGGTGCCTGCCTACTTCAA 3’ and HSP70 rev: 5’CCGCCCATGCTCTGGTACATC 3’, generating a 1300 bp fragment [19]. The reaction was prepared to a final volume of 25 μL using 5μL of DNA template, 0.75 μl of MgCl_2_ at 50 mM, 0.5 μl of dNTP mix at 10 mM (Promega), 1.5 μl of DMSO 5.0% (Invitrogen), 0.25 μl of Taq DNA polymerase platinum^®^ at 5U/μl (Invitrogen), 1μl of HSP70 primer for at 10 pmol and 1μl of primer HSP70 rev at 10 pmol (IDT, prodimol). The DNA was amplified in an automatic thermocycler (Veriti—Applied Biosystems). The amplification conditions were described by Garcia et al., 2004 [19]. Every assay included DNA extraction negative and positive controls and PCR negative and positive controls. The amplified products were visualized on a 2.0% agarose gel stained with ethidium bromide. Samples that had a specific band at 1300 bp were subjected to digestion using the enzyme *Hae*III for analysis of restriction fragment length polymorphisms [19]. The digestion reaction was prepared to a final volume of 15 μL, containing 1μL of HaeIII (New England) (10 U/μL), 1.5 μL enzyme buffer and 12.5 μL amplified product and the mixture was incubated at 37°C for 2 hours. The restriction profiles were analyzed in 4% agarose and compared with reference strains of *Leishmania amazonensis* (IFLA/BR/67/PH8),*L*. *braziliensis* (MHOM/BR/75/M2903), *L*. *infantum* (MHOM/BR/74/PP75) and *L*. *guyanensis* (MHOM/BR/75/M4147).

## Results

**Fig 1:**
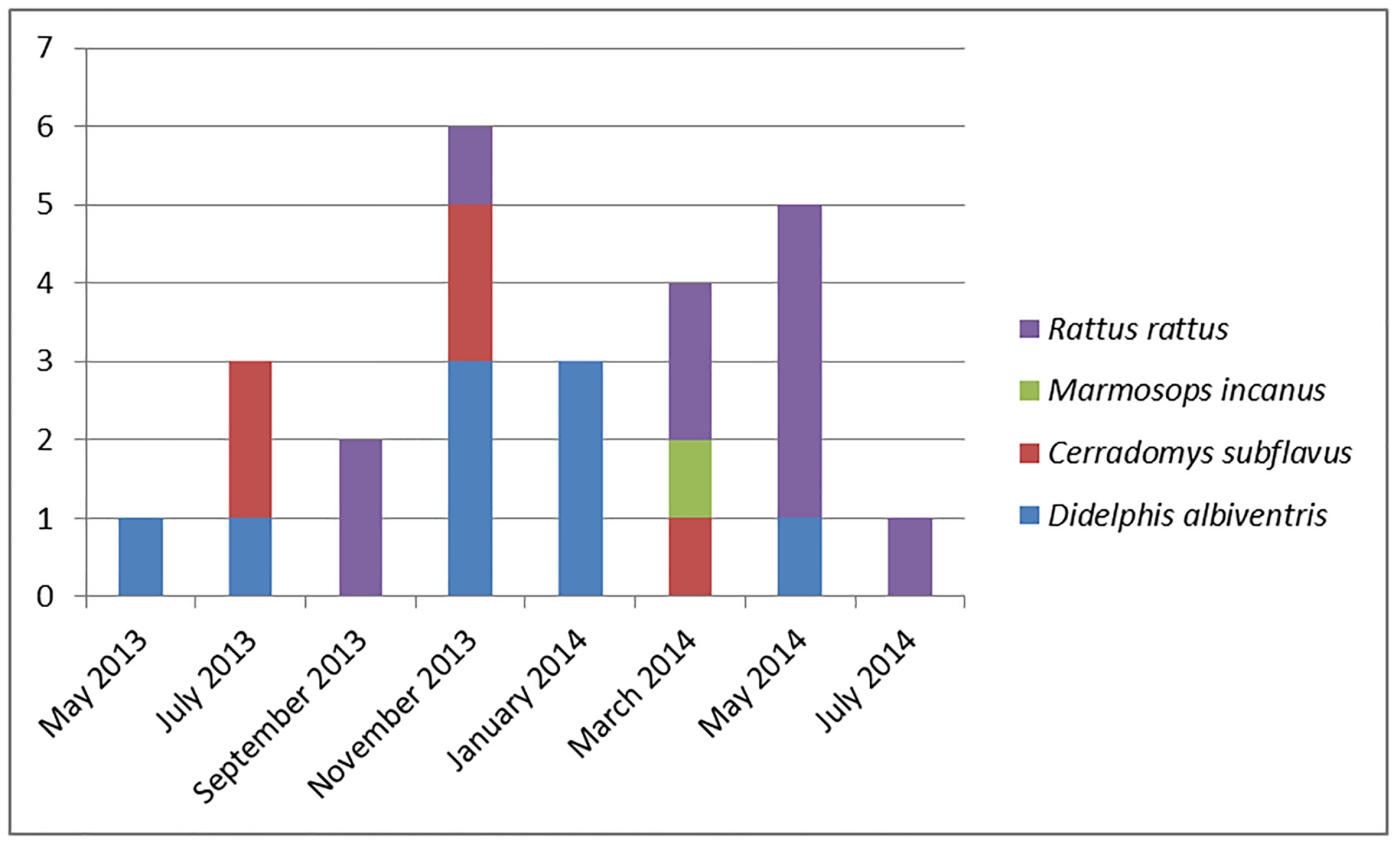
Number of specimens captures by species and campaign (May 2013 to July 2014) in Casa Branca, Brumadinho, MG, Brazil.

A total of 25 small mammals were collected, including 15 rodents of the species *Rattus rattus and Cerradomys subflavus*, and 10 marsupials of the species *Didelphis albiventris* and *Marmosops incanus*. *Rattus rattus* was the most captured species with 10 specimens (40%), followed by *Didelphis albiventris* with 9 (36%), *Cerradomys subflavus* with 5 (20%) and *Marmosops incanus* with one specimen (4%) (Fig 1, S1 Table).

**Fig 2:**
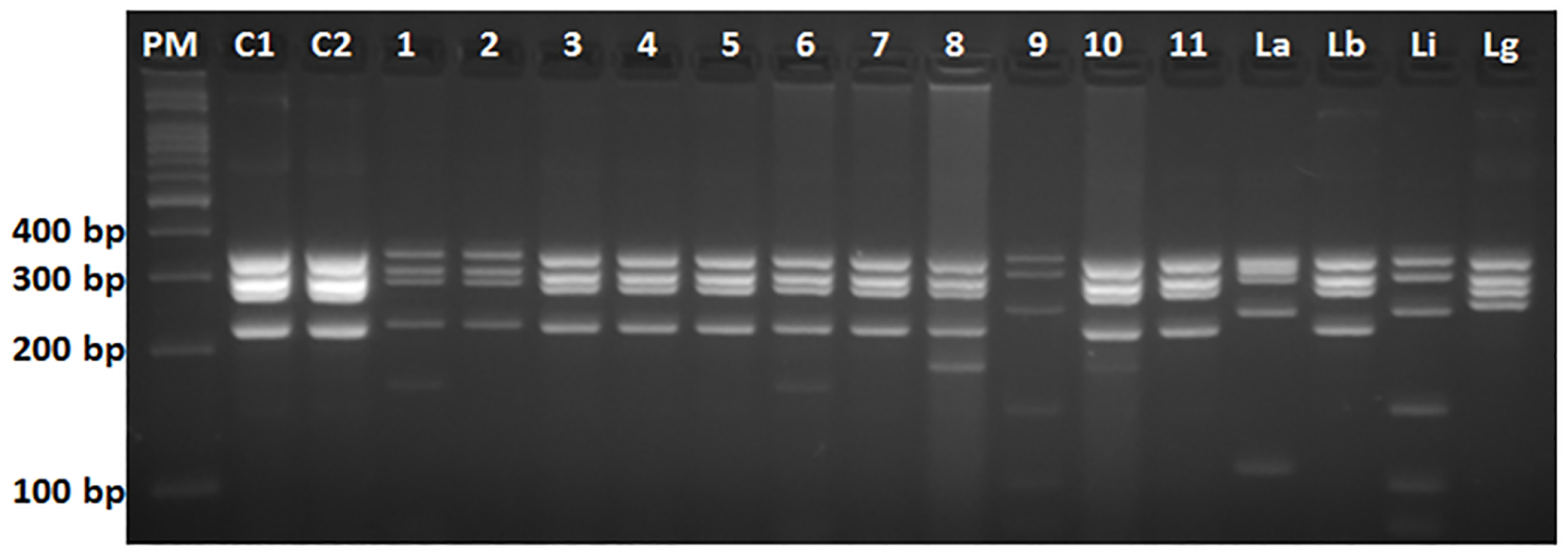
Results of PCR RFLP *hsp*70. PM: 100bp molecular weight marker. C1 and C2:DNA samples of isolated parasites. 1 to 11: DNA samples of small mammals. La: *L*. *(L*.*) amazonensis* (IFLA/BR/1967/PH8); Lb: *L*. *(Viannia) braziliensis* (MHOM/BR/1975/M2903); Li: *L*. *(L*.*) infantum* (MHOM/BR/1974/PP75); and Lg: *L*. *(V*.*) guyanensis* (MHOM/BR/1975/M4147).

*Leishmania* sp. parasites were isolated in culture medium from liver and spleen samples from the same *R*. *rattus* specimen. DNA samples from these isolates (C1 liver and C2 spleen) were subjected to PCR-RFLP hsp70 and exhibited a profile compatible with the *L*. *braziliensis*. This same pattern was observed in the positive tissue samples of this individual (Fig 2).

The positivity indexes for samples by tissue and by animal are shown in Table 1. PCR of *hsp*70 was positive in 20% of mammal captured, among marsupials (2/10) and rodents (3/15). *Leishmania* fragment of *hsp*70 gene was detected in samples of liver (4), spleen (3), ear skin (2) and tail skin (1). None of the samples of bone marrow were considered positive.

**Table 1:** PCR positivity for *hsp*70 in tissue samples of small mammals captured in Casa Branca, Brumadinho, Minas Gerais, Brazil, in the period from May 2013 to June 2014.

			Positive tissues					
Species	Specimens captured	Positive animals (%)	Liver	Spleen	Tail skin	Ear skin	Bone marrow	Total +/n(%)
***Didelphis albiventris***	9	2 (22,2)	2	1	0	1	0	4 / 45 (8,8)
***Marmosops incanus***	1	0	0	0	0	0	0	0/5(0)
***Cerradomys subflavus***	5	1(20)	1	0	0	1	0	2/25(8)
***Rattus rattus***	10	2 (20)	1	2	1	0	0	4/50(8)
Total	25	5 (20)	4	3	1	2	0	10/125(20)

*Leishmania braziliensis* was identified by PCR-RFLP *hsp70* in samples of two specimens of *D*. *albiventris*, one specimen of *C*. *subflavu*s and one specimen of *R*. *rattus*, *and Leishmania infantum* was identified in one sample of a *R*. *rattus* (Fig 2).

## Discussion

Studies show that 60% of human disease may have a zoonotic origin [20, 21] and among these are leishmaniases. The role played by different mammalian hosts in the transmission cycle of the majority of species of *Leishmania* remains poorly understood, which reinforces the need for studies that document the occurrence of these parasites in mammals other than those classically described as reservoirs [22].

In recent years, there has been an increase in the number of cases of both CL and VL in the municipality of Brumadinho, and Casa Branca contributed significantly. The control measures for leishmaniases in Brumadinho are performed in a sporadic and emergency manner. The consequence of this lack of prevention actions and continuous control may be an increase in the incidence of leishmaniases and probably a future increase in mortality by VL and morbidity by CL in the municipality. Therefore, the study of aspects of the transmission chain are crucial to the development of preventive and control measures for the reduction of the spread of leishmaniasies in the municipality. This work investigated the occurrence of *Leishmania* infections in small mammals and the results indicate that *Leishmania braziliensis* and *Leishmania infantum* circulate among the small mammals that make up the dominant fauna of Casa Branca.

In this study, the most sampled mammal was *R*. *rattus*, a highly synanthropic terrestrial rodent, which also has a great ability to climb, and so is frequently found in the ceilings and walls of houses. Two specimens of this species were infected, one by *L*. *(L*.*) infantum* and the other by *L*. *(V*.*) braziliensis*. From the latter animal, it was possible to isolate the parasite, which was the first record of *L*. *(V*.*) braziliensis* isolated from a *R*. *rattus* in Minas Gerais. This animal was captured in a peridomicile area of a residence that was raising domestic animals very close to the house, and possessed an accumulation of organic matter, both of which are factors that contribute to the attraction of rodents, and are favorable for the development of phlebotomine vectors. In the residence where this animal was captured there was previous record of canine leishmaniasis. A similar result was observed by Ferreira et al [8] in Belo Horizonte, which borders the municipality of Brumadinho. This study found DNA of *L*. *braziliensis* and *L*. *infantum* in specimens of *R*. *rattus* collected in peridomicile areas of residences.

This species has been found infected by *L*. *braziliensis* in other regions of Brazil [23, 24, 25, 26] and Brandão-Filho et al [10] isolated *L*. *braziliensis* from a specimen of *R*. *rattus* from the state of Pernambuco. Andrade et al [27], studied the experimental infection by *L*. *braziliensis* in two species of wild rodents and in *R*. *rattus*. The ability of the three species to maintain the infection as well as to infect specimens of *Lutzomyia longipalpis* was observed. These data reinforce the hypothesis that the main reservoirs for *L*. *(V*.*) braziliensis* are rodents.

The second most collected species was the opossum *Didelphis albiventris*, which also provided two specimens infected with *L*. *(V*.*) braziliensis*. This marsupial has been previously found infected by this parasite in other regions of Brazil [24, 10, 28, 7, 8], and is of epidemiological importance because it is associated with habitats near human residences to feed on poultry [29] *Cerradomys subflavus*, a rodent that inhabits forest formations and natural open habitats, had one individual specimen infected by *L*. *(V*.*) braziliensis*. There are previous reports of this species being infected by *L*. *(Viannia)* sp. [30] and by a parasite belonging to the *L*. *braziliensis* complex [26, 8]. These native species, *C*. *subflavus* and *D*. *albiventris* are usually captured near human residences and in natural habitats.

The composition and diversity of animal species can be altered by the destruction of the natural environment and may modify the dynamics of the transmission of pathogens that cause zoonoses such as *Leishmania* spp. [31]. In the study area the main environmental impacts come from the intensification of the economic activities. The real state pressure, mining activities and the establishment of agricultural areas are responsible for the impacts, especially the degradation of springs, the triggering of erosive processes, the silting up of streams, deforestation, among others. On the other hand, there are large green areas important to balance the environmental impacts of the above mentioned activities. Therefore the mammal species with synanthropic habits may play an important role in the connection between these different environmental scenarios acting as bridge for the circulation of *Leishmania* species between natural and anthropic habitats.

For human diseases transmitted by insects it has been observed that vectors may be attracted by a variety of hosts, not humans. The vector-based transmission system is dependent on several factors that are critical to understanding the transmission dynamics in different environments [31]. The finding of several species of mammals infected by *L*. *braziliensis* suggests that this species belongs to the multi-host parasite group. According to Ostfeld & Keesing [31], the dynamics of multi-host parasites can be extremely complex and may be influenced by factors intrinsic (genetic diversity, frequency) or extrinsic (environmental changes) to the host.

The finding of two species of *Leishmania* infecting different species of mammals reflects the dynamism and complexity of the transmission cycles of these parasites in the study area. In addition, the capture of infected animals near residences emphasizes the possible relationships of human cases to these animals, and points to the need for more studies to contribute to defining measures to prevent the transmission of species of *Leishmania*.

## Supporting information

S1 Table Table with informations about the animals captured in Brumadinho, Minas Gerais, Brazil. (PDF)
