# Supplementary material for: Detection of Leishmania spp in silvatic mammals and isolation of Leishmania (Viannia) braziliensis from Rattus rattus in an endemic area for leishmaniasis in Minas Gerais State, Brazil
Source: PLoS One. 2017 Nov 27;12(11):e0187704. doi: 10.1371/journal.pone.0187704 (PMC5703529; doi:10.1371/journal.pone.0187704)
Supplement: S1 Table — (PDF) [file pone.0187704.s001.pdf]

				hsp 70 PCR				
Number	Specie	Date of capture	Area of capture	Liver	Spleen	Ear skin	Tail skin	Bone marrow
1	*Didelphis albiventris*	May 2013	Peridomicile	Positive	Negative	Negative	Negative	Negative
2	*Cerradomys subflavus*	June 2013	Peridomicile	Positive	Negative	Negative	Positive	Negative
3	*Didelphis albiventris*	June 2013	Peridomicile	Positive	Positive	Positive	Negative	Negative
4	*Cerradomys subflavus*	June 2013	Peridomicile	Negative	Negative	Negative	Negative	Negative
5	*Rattus rattus*	September 2013	Peridomicile	Positive	Positive	Negative	Negative	Negative
6	*Rattus rattus*	September 2013	Peridomicile	Negative	Positive	Negative	Positive	Negative
7	*Rattus rattus*	November 2013	Peridomicile	Negative	Negative	Negative	Negative	Negative
8	*Didelphis albiventris*	November 2013	Peridomicile	Negative	Negative	Negative	Negative	Negative
9	*Didelphis albiventris*	November 2013	Peridomicile	Negative	Negative	Negative	Negative	Negative
10	*Didelphis albiventris*	November 2013	Peridomicile	Negative	Negative	Negative	Negative	Negative
11	*Cerradomys subflavus*	November 2013	Peridomicile	Negative	Negative	Negative	Negative	Negative
12	*Cerradomys subflavus*	November 2013	Peridomicile	Negative	Negative	Negative	Negative	Negative
13	*Didelphis albiventris*	January 2014	Peridomicile	Negative	Negative	Negative	Negative	Negative
14	*Didelphis albiventris*	January 2014	Peridomicile	Negative	Negative	Negative	Negative	Negative
15	*Didelphis albiventris*	January 2014	Peridomicile	Negative	Negative	Negative	Negative	Negative
16	*Cerradomys subflavus*	March 2014	Peridomicile	Negative	Negative	Negative	Negative	Negative
17	*Marmosops incanus*	March 2014	Peridomicile	Negative	Negative	Negative	Negative	Negative
18	*Rattus rattus*	March 2014	Peridomicile	Negative	Negative	Negative	Negative	Negative
19	*Rattus rattus*	March 2014	Peridomicile	Negative	Negative	Negative	Negative	Negative
20	*Didelphis albiventris*	May 2014	Peridomicile	Negative	Negative	Negative	Negative	Negative
21	*Rattus rattus*	May 2014	Peridomicile	Negative	Negative	Negative	Negative	Negative
22	*Rattus rattus*	May 2014	Peridomicile	Negative	Negative	Negative	Negative	Negative
23	*Rattus rattus*	May 2014	Peridomicile	Negative	Negative	Negative	Negative	Negative
24	*Rattus rattus*	May 2014	Peridomicile	Negative	Negative	Negative	Negative	Negative
25	*Rattus rattus*	July 2014	Peridomicile	Negative	Negative	Negative	Negative	Negative
